# Linkage mapping and genomic prediction of grain quality traits in tropical maize (*Zea mays* L.)

**DOI:** 10.3389/fgene.2024.1353289

**Published:** 2024-02-21

**Authors:** Noel Ndlovu, Rajashekar M. Kachapur, Yoseph Beyene, Biswanath Das, Veronica Ogugo, Dan Makumbi, Charles Spillane, Peter C. McKeown, Boddupalli M. Prasanna, Manje Gowda

**Affiliations:** ^1^ Agriculture and Bioeconomy Research Centre, Ryan Institute, University of Galway, Galway, Ireland; ^2^ International Maize and Wheat Improvement Center (CIMMYT), Nairobi, Kenya; ^3^ University of Agricultural Sciences, Dharwad, Karnataka, India

**Keywords:** grain quality traits, quantitative trait loci, genomic prediction, tropical maize, sub-Saharan Africa

## Abstract

The suboptimal productivity of maize systems in sub-Saharan Africa (SSA) is a pressing issue, with far-reaching implications for food security, nutrition, and livelihood sustainability within the affected smallholder farming communities. Dissecting the genetic basis of grain protein, starch and oil content can increase our understanding of the governing genetic systems, improve the efficacy of future breeding schemes and optimize the end-use quality of tropical maize. Here, four bi-parental maize populations were evaluated in field trials in Kenya and genotyped with mid-density single nucleotide polymorphism (SNP) markers. Genotypic (G), environmental (E) and G×E variations were found to be significant for all grain quality traits. Broad sense heritabilities exhibited substantial variation (0.18–0.68). Linkage mapping identified multiple quantitative trait loci (QTLs) for the studied grain quality traits: 13, 7, 33, 8 and 2 QTLs for oil content, protein content, starch content, grain texture and kernel weight, respectively. The co-localization of QTLs identified in our research suggests the presence of shared genetic factors or pleiotropic effects, implying that specific genomic regions influence the expression of multiple grain quality traits simultaneously. Genomic prediction accuracies were moderate to high for the studied traits. Our findings highlight the polygenic nature of grain quality traits and demonstrate the potential of genomic selection to enhance genetic gains in maize breeding. Furthermore, the identified genomic regions and single nucleotide polymorphism markers can serve as the groundwork for investigating candidate genes that regulate grain quality traits in tropical maize. This, in turn, can facilitate the implementation of marker-assisted selection (MAS) in breeding programs focused on improving grain nutrient levels.

## 1 Introduction

Maize (*Zea mays*. L) ranks among the most prominent coarse cereal crops on a global scale, alongside rice and wheat (Erenstein et al., 2021). It is a widely cultivated crop, spanning over 170 countries and covering a vast area of 197 million hectares (FAOstat, 2022). In 2020, maize cultivation spanned approximately 43 million ha in Sub-Saharan Africa (SSA), contributing to a substantial production of around 90 million metric tonnes. On a continental scale, Africa accounts for approximately 20.9% of the total global maize cultivation area. However, its contribution to global maize production is comparatively lower (∼7.4%) (Prasanna et al., 2021). Indeed, maize yield growth varies across regions, with Africa lagging at 1.3%, while the United States of America leads at 2.0%, followed by Asia at 1.8% (Erenstein et al., 2022). Despite the widely reported low productivity per unit area, maize is the major source of food for more than 80% of the population in SSA (Prasanna et al., 2021) and meets more than 30% of their calorie requirement (Goredema-Matongera et al., 2021). Maize cuisine in this region is remarkably diverse, encompassing six distinct categories: whole-maize foods, wet-ground foods, snacks, bread, maize sourdough, and dumplings (Ekpa et al., 2018). Within this spectrum, stiff porridge, prepared by slowly adding maize flour to boiling water until it reaches the desired thickness - called *ugali* in Kenya and Tanzania, *nshima* in Zambia, *sadza* in Zimbabwe, and *mealiepap* in South Africa - stands out as a widely favoured culinary choice (De Groote and Kimenju, 2012). This staple, akin to rice in Asian cuisines, holds significant prominence and is widely consumed.

The substantial dependence of the SSA population on maize-based foods comes with inherent drawbacks, given that a significant proportion of the accessible maize varieties lack adequate levels of essential minerals. Ranum et al. (2014) indicated that while tropical maize is characterized by a high carbohydrate content (∼72%), its grain protein content is modest, ranging from 9% to 10% and its fat content is around 4%. The region’s reliance on maize for sustenance, particularly among low-income farming communities facing malnutrition, amplifies the urgency of addressing maize yield stagnation. Maize production in these communities is globally the lowest, standing at 2.1 t/ha. This can be attributed to various factors, with a considerable portion linked to the restricted availability of improved hybrid seeds (Quarshie et al., 2021). Other contributing factors include low-input agricultural practices, such as the application of only 8.0 kg of fertilizers per ha compared to the global average of 137 kg/ha (Das et al., 2019), small land holdings, limited access to mechanisation, sub-optimal post-harvest management, and challenges related to biotic and abiotic stresses. Some of these challenges impacting maize production in SSA have a negative effect on grain quality composition, notably the critical issue of low soil nitrogen stress. The application of nitrogenous fertilizer in smallholder farming systems within this region is severely constrained, reported by FAOSTAT (2023) at 4%. The effect of soil nitrogen on maize grain yield, composition and quality has been extensively investigated by numerous researchers (Worku et al., 2012; Biswas and Ma, 2016; Zhang et al., 2020; Ertiro et al., 2022; Ndlovu et al., 2022; Hammad et al., 2023). Although findings have varied, there is a consensus on the existence of genotypic differences in grain yield and composition among tropical maize genotypes grown under diverse management conditions.

The integration of genomic tools with field-based breeding techniques holds promise for enhancing nutrient composition in maize grain. Among these genomic techniques, quantitative trait loci (QTL) mapping stands out as a classical method for identifying genomic regions associated with traits of interest (Yang et al., 2020; Goering et al., 2021; Ren et al., 2022), even in the absence of prior genetic knowledge about the specific trait(s). Several studies have identified major QTLs associated with starch, protein, and oil contents in maize grain. In 275 recombinant inbred lines (RILs), Lu et al. (2022) identified 11 QTLs affecting kernel protein content some linked to the *Zm00001d002625* gene which encodes an S-adenosyl-L-methionine-dependent methyltransferase superfamily protein. Guo et al. (2013) identified nine unconditional QTLs across all chromosomes (excluding chr 3 and 7) for oil content and eight unconditional QTLs distributed across all chromosomes except chromosomes 4 and 8 for starch content. Liu et al. (2008) identified a total of eighteen QTLs for grain quality traits across diverse soil nitrogen regimes and locations. Among these, seven QTLs were associated with oil content, six with protein content, and five with starch content. Ndlovu et al. (2022) also identified multiple QTLs for maize grain quality traits using multiple biparental populations under different soil nitrogen levels. Application of genomic selection for different traits in maize revealed moderate to high prediction accuracies (Gowda et al., 2018; 2021; Ertiro et al., 2020; Beyene et al., 2021). Ndlovu et al. (2022) applied genomic selection on maize grain quality traits under optimum and low soil N management and observed moderate to high accuracies in both diversity panel and biparental populations. In the preceding studies, empirical evidence has substantiated that the integration of traditional breeding methodologies with linkage mapping, coupled with genomic selection, markedly enhances the efficiency of improving nutritional quality traits in maize.

Though many studies reported several genomic regions associated with grain quality traits, additional sources of genetic variation exist in unexplored maize populations. On the flip side, the rapid acquisition of high-quality phenotypic data to facilitate genomic analyses for grain-quality traits continues to be a resource-demanding endeavour. Indeed, the conventional phenotyping methods for assessing grain quality traits are characterized by their labour-intensive nature and time-consuming processes. On a positive note, the expanded utilization of Near Infrared Reflectance Spectroscopy (NIRS) has enhanced the capabilities of nutritional profiling studies for maize grain. NIRS is a fast, reliable, and non-destructive method that is being employed in plant phenotyping assessment of maize kernel starch, protein and oil content (Ndlovu et al., 2022). In this study, we used NIRS to measure the main nutritional quality traits in four bi-parental maize populations and used mid-density single nucleotide polymorphism (SNP) markers for both linkage mapping and genomic prediction. Our study was designed to accomplish the following objectives: (i) to assess the genetic variation in grain quality traits (i.e., protein, oil and starch) among tropical maize populations; (ii) to identify significant QTLs associated with grain quality traits in tropical maize populations tested across multiple environments, and (iii) to assess the potential of utilizing genomic selection for the improvement of grain quality traits in tropical maize.

## 2 Materials and methods

### 2.1 Plant materials

One doubled haploid (DH) and three F_3_ tropical maize populations comprising 110, 271, 333 and 158 lines, respectively, were developed from four bi-parental crosses (Table 1). The populations used in this study were also used for mapping agronomic traits in our earlier studies (Ertiro et al., 2020; Ndlovu et al., 2022; Kimutai et al., 2023). All the parental lines we used in this study exhibited variations in grain quality traits (oil, protein and starch content) and are adapted to mid-altitude regions (1,000–1,500 m above sea level (MASL)) of SSA. Studied populations were test-crossed with an appropriate tester from the opposite heterotic group for phenotypic evaluations. The performance of parental lines and selected commercial hybrids (Supplementary Table S1) and the progenies for each population are listed (Supplementary Table S2) for all quality traits. Each population along with its parents were planted at the Kiboko Maize Research Station, Kiboko, Kenya. Kiboko Maize Research Station is situated between 37.7235°E longitude and 2.2172°S latitude, at an elevation of 975 MASL. The annual temperature ranges from 16.0°C to 33.6°C, and the rainfall varies from 545 to 629 mm annually across the two studied seasons. This location lies in a hot, semi-arid region and the soils are well-drained, dark reddish brown to dark red, friable sandy clay to clay (Acri-Rhodic Ferrosols) developed from undifferentiated basement system rocks, predominantly banded gneisses (Ertiro et al., 2022). The DH pop 1 and F_3_ pop 2 were evaluated at Kiboko in the main rainy season for two seasons, while F_3_ pop 3 and F_3_ pop 4 were evaluated for one season.

**TABLE 1:** Details of the maize populations used in this study.

Population	Pedigree	Population size
DH Population 1	CML494×CML550	110
F_3_ Population 2	CKL05017×CML536	276
F_3_ Population 3	CML494×CML550	315
F_3_ Population 4	VL081452×VL058589	158

### 2.2 Field trial and data collection

All four biparental populations were evaluated using an alpha lattice incomplete block design with two replications. Single row plots, measuring 5 m long at a row spacing of 0.75m, were sown. Trial plots were top-dressed with urea fertilizer at the rate of 138 kg N per hectare 3 weeks post-planting. All trials were irrigated as required to avoid any moisture stress. Trials were kept weed-free and other established standard agronomic practices were followed. Data pertaining to the target traits were systematically collected by selecting ten plants from the midsection of the plot rows. This included plant height (PH, centimetres), anthesis date (AD, days), anthesis silking interval (ASI, days), ear height (EH, centimetres) and ear position (EPO, ratio of EH/PH). For grain yield (GY) assessment, the shelled grains were quantified in kilograms (kg) and subsequently converted to tons per hectare, considering a moisture content of 12.5% (GY in t/ha).

Grain texture was measured on a 1 to 5 scale (where 1 = flint, 2 = semi-flint, 3 = intermediate, 4 = semi-dent and 5 = dent). Kernel weight (Kwt) was determined by measuring 100 randomly selected seeds per line/family per replication, with measurements recorded in grams. Following harvest, seeds for each genotype were separated to facilitate grain nutrient analyses. Grain quality traits, specifically oil, protein, and starch contents, were quantified using a FOSS Infratec TM 1241. The analysis involved 500 g samples of maize grain obtained from each plot, and the results were reported as a percentage of whole grain. Five 100-g subsamples were assayed and the mean reading for each parameter was reported per plot. The reflectance spectra were collected in a range of 400–2,500 nm with 10 nm intervals in the near-infrared reflectance (NIR) region.

Analyses of variance (ANOVA) for grain traits, including oil content, protein content, starch content, kernel weight, and grain texture, within each biparental population across different seasons were performed using the META-R (Alvarado et al., 2020) and ASREML-R (Gilmour et al., 2002). Best Linear Unbiased Estimators (BLUEs) were computed utilizing a mixed model, wherein genotype entries were treated as fixed effects, while the remaining terms were treated as random. In the estimation of broad-sense heritability, all terms were considered as random effects. Broad sense heritability was estimated by the formula:

$$h^{2}=\sigma_{G}^{2}/\left(\sigma_{G}^{2}+\sigma_{\text{GE}}^{2}/E+\sigma_{e}^{2}/\text{Er}\right)$$

Where **σ** ^**2**^_**G**_ is the genotypic variance, **σ** ^**2**^_**GE**_is the genotypic by environment interaction (GEI), **σ** ^**2**^_**e**_ is the error variance, **E** is the number of environments or seasons, and **r** is the number of replications in each trial. The phenotypic and genotypic correlations among traits were evaluated as described by Hirel et al. (2007).

### 2.3 Genotypic analysis

DNA was extracted from bulked young leaves of the studied tropical maize lines following the CTAB Method (CIMMYT, 2005). Genotyping of the F_3_ populations was performed using the Illumina Maize SNP1500 Bead Chip, which utilizes evenly spaced SNPs to comprehensively cover the maize genome (Ganal et al., 2011). The above task was performed at the LGC genomic labs in the United Kingdom (https://www.lgcgroup.com/genotyping/). In the case of the tested DH population, lines were genotyped using the Genotyping-by-Sequencing (GBS) approach. The obtained data underwent filtration using TASSEL software, with criteria set at > 0.10 Minor Allele Frequency (MAF), <5% heterozygosity, and a minimum count of 90% of the total size (Bradbury et al., 2007; Sitonik et al., 2019). In all the populations, homozygous marker loci for both parents and uniformly distributed polymorphic markers between parents were retained. Linkage maps in all four populations were constructed using QTL IciMapping version 4.1 software (Meng et al., 2015). After removing the distorted markers, finally, we used 1,007, 452, 231, and 387 high-quality SNPs in DH pop 1, F_3_ pop 2, F_3_ pop 3 and F_3_ pop 4, respectively. In brief, the linkage map was constructed by using these SNPs, and by selecting the most significant markers using stepwise regression. A likelihood ratio test was used to calculate the logarithm of odds (LOD) for each marker at a score of >3 with a 30 cM maximum distance between two loci. The recombination frequency between linked loci was transformed into cM (CentiMorgan’s units) using Kosambi’s mapping function (Kosambi, 1944).

For each population, best linear unbiased predictors (BLUPs) across seasons were used to detect QTLs based on Inclusive interval mapping (ICIM). The quantification of phenotypic variation attributed to individual QTLs and the cumulative variation explained by the aggregate presence of all QTLs was conducted. QTL names were constructed by starting with the letter ‘q' to indicate QTL, followed by an abbreviation of the trait name, the corresponding chromosome number, and the marker position (Ribaut et al., 1997).

Genomic prediction (GP) analysis was performed in R program version 4.2.1 (R Core Team 2023). GP was applied to each F_3_ and DH population to find out the prediction accuracy of grain quality traits. This was done using the RR-BLUP model (Zhao et al., 2012; Crossa et al., 2017). BLUEs across seasons for each of the studied populations were used for the analysis. In GP analysis, polymorphic SNPs between the parents of each population, comprising 1,007, 452, 231, and 387 SNPs in DH pop 1, F_3_ pop 2, F_3_ pop 3, and F_3_ pop 4, respectively, were used. A five-fold cross-validation approach was employed, specifically utilizing a ‘within population’ strategy where both training and estimation sets originated from within each bi-parental population. For each trait in each population, 100 iterations were performed to divide the data into training and estimation sets.

## 3 Results

### 3.1 Phenotypic analyses

**FIGURE 1:**
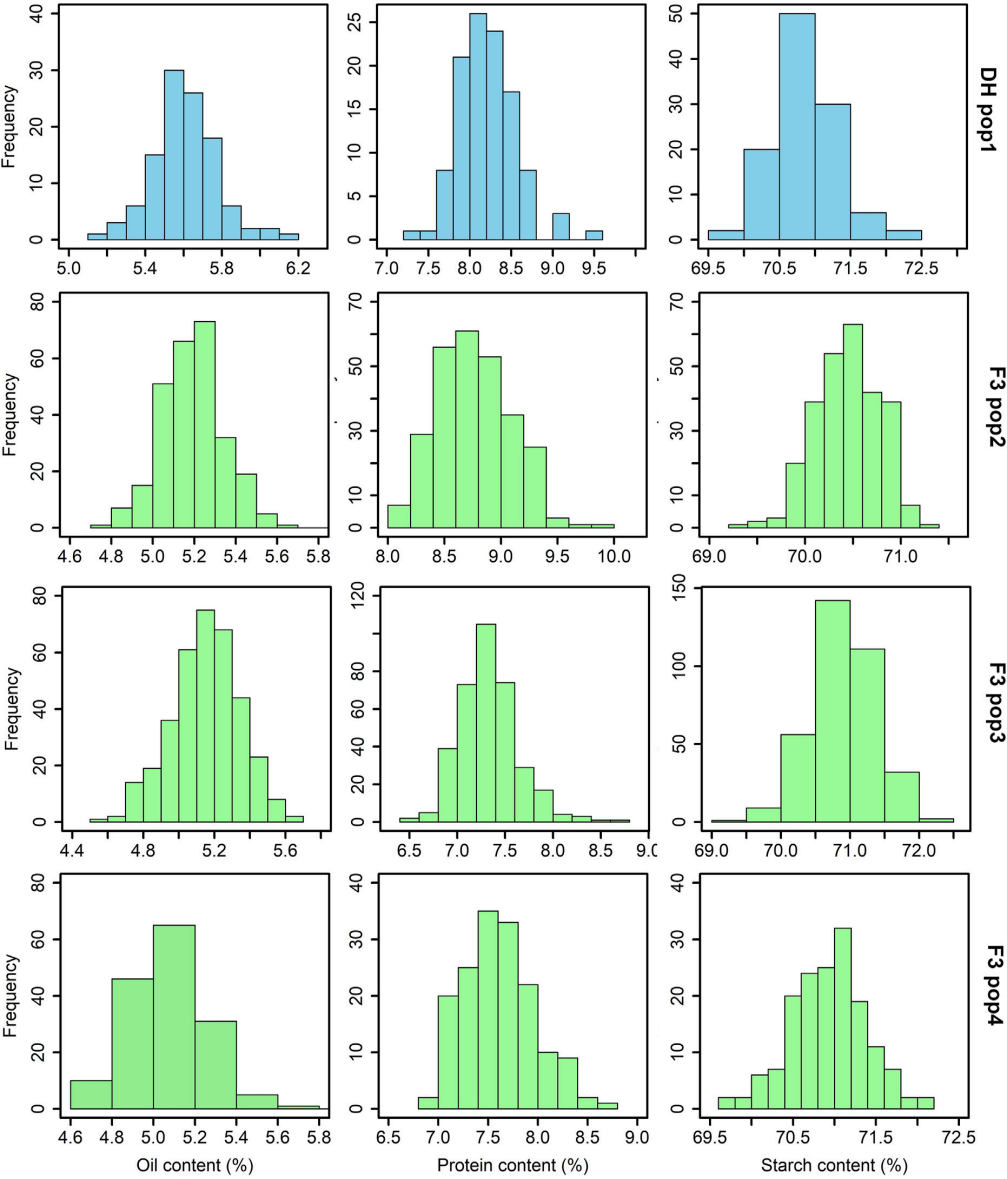
Phenotypic distribution for grain quality traits evaluated across DH and F_3_ tropical maize populations. The sky blue and light green colour plots represent the grain quality trait measurements for DH and F_3_ populations, respectively. DH pop 1 = CML494×CML550; F_3_ pop 2 = CKL05017×CML536; F_3_ pop 3 = CML494×CML550; and F_3_ pop 4 = VL081452×VL05858.

The analysis of grain quality traits revealed significant variability within the studied tropical maize populations for each trait. The distribution of these traits closely followed the expected normal distribution pattern, as demonstrated by the histogram plots (Figure 1). Across the DH and F_3_ populations, the mean protein content varied from 6.8%–10%, oil content from 4.5%–6.2% and starch content ranged from 69.5%–72.5%. Among these populations, F_3_ pop 2 had the highest protein content (10%) followed by DH pop 1 (9.6%). F_3_ pop 3 and F_3_ pop 4 recorded grain protein levels <9%. For oil content, DH pop 1 (6.2%) exhibited the highest values, followed by F_3_ pop 4 (5.8%). For starch content, the highest levels were recorded in DH pop 1 and F_3_ pop 3 (both with 72.5%).

Genotypic variance (σ^2^_G_) was significant at *p* ≤ 0.05 for grain quality traits (oil, protein, starch content, kernel weight and grain texture) across all the studied genotypes (Table 2). Genotype-environment interaction effects (σ^2^_G×E_) were significant for protein and starch content in DH pop 1 and F_3_ pop 2. In general, kernel weight consistently exhibited elevated coefficients of variation (CVs) within both DH and F_3_ populations. When comparing the same trait across the studied populations, the CVs for the F_3_ pop 4 were marginally higher than those for other populations. Nonetheless, substantial variations across the genotypes and traits were still evident.

**TABLE 2:** Estimates of means, components of genotypic (σ^2^_G_), genotype × environment interaction (σ^2^_G×E_), error variances (σ^2^_e_) and heritability (h^2^) for four biparental populations evaluated at Kiboko in Kenya for grain quality traits (protein content, starch content, oil content and grain texture).

Populations	Grain quality traits			
	Oil content	Protein content	Starch content	Texture/Kernel weight^1^
DH pop 1 - CML494×CML550				
σ^2^_G_	0.02*	0.03*	0.04**	4.37*
σ^2^_G×E_	0.01	0.03*	0.02*	-
σ^2^_e_	0.03	0.24	0.41	21.30
h^2^	0.68	0.29	0.26	0.31
LSD_5%_	0.22	0.38	0.49	5.07
CV (%)	3.23	5.94	1.01	7.06
F_3_ pop 2 - CKL5017×CML536				
σ^2^_G_	0.02*	0.04**	0.04**	0.07**
σ^2^_G×E_	0.01	0.06**	0.01*	0.04*
σ^2^_e_	0.04	0.11	0.21	0.19
h^2^	0.58	0.42	0.42	0.67
LSD_5%_	0.22	0.44	0.46	0.43
CV (%)	3.66	3.85	0.69	19.76
F_3_ pop 3 - CML494×CML550				
σ^2^_G_	0.02*	0.04*	0.07**	3.47**
σ^2^_e_	0.03	0.09	0.27	14.84
h^2^	0.57	0.47	0.34	0.35
LSD_5%_	0.26	0.42	0.60	3.11
CV (%)	3.55	4.18	0.73	7.33
F_3_ pop 4 - VL081452×VL058589				
σ^2^_G_	0.02*	0.04*	0.04*	11.50**
σ^2^_e_	0.04	0.15	0.22	30.42
h^2^	0.50	0.35	0.27	0.43
LSD_5%_	0.30	0.46	0.51	7.38
CV (%)	4.16	5.17	0.80	7.89

CV- coefficient of variation, LSD-least significant difference, *, ** significant at *p*=0.05 and 0.01 level, respectively.^1^ Grain texture was recorded in F_3_ pop 2 whereas kernel weight was recorded in the other three populations.

Across the segregating populations, the highest and lowest values of broad sense heritabilities were observed for oil content (0.68) and starch content (0.26) in DH pop 1. Overall broad-sense heritabilities ranged from low to high for all grain quality traits studied–with oil content (0.68) and grain texture (0.67) having the highest values. Generally, oil content (0.50–0.68) had moderate to high broad-sense heritabilities across the studied populations. Whereas protein (0.29–0.47) and starch (0.26–0.42) contents, on the other hand, recorded low to moderate values of heritability across the populations. Likewise, for kernel weight trait the heritability values ranged from low (0.31 in DH pop 1) to high (0.43 in F_3_ pop 4).

**FIGURE 2:**
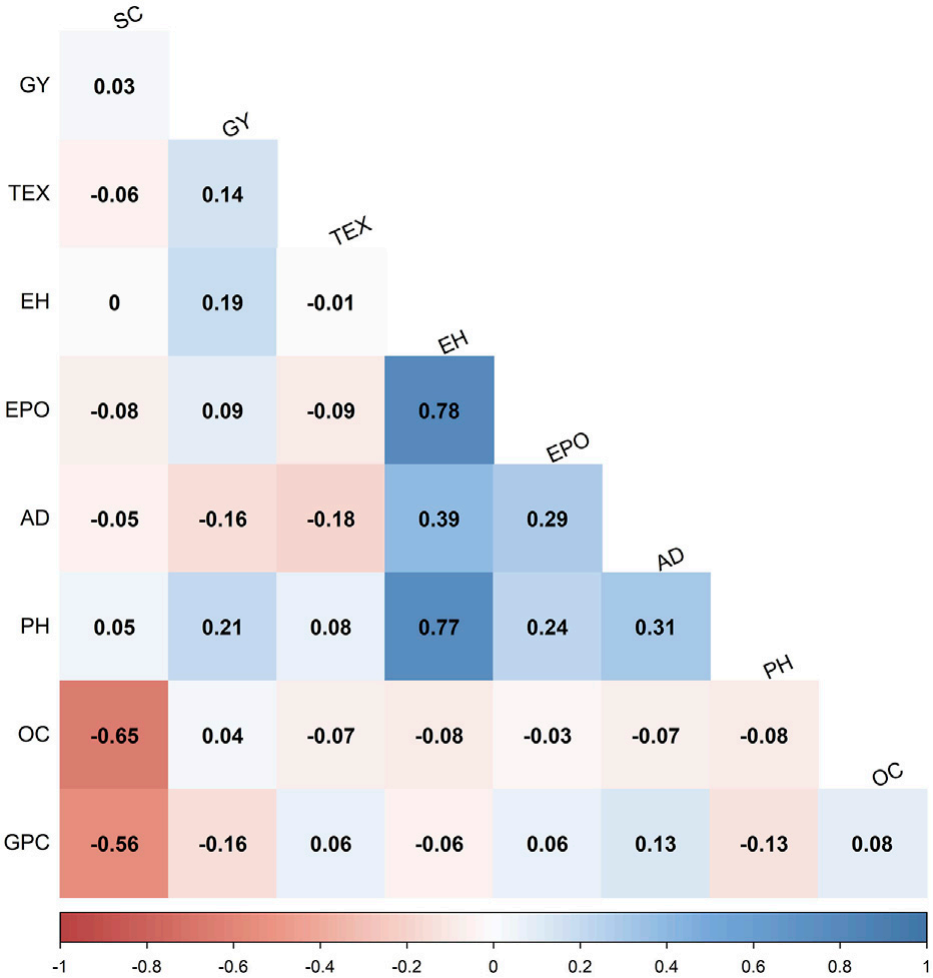
Phenotypic correlations of grain quality and other agronomic traits evaluated in F_3_ pop 2 (CKL5017×CML536). The correlation values < 0.11 are not significant at *p* < 0.05. GPC = grain protein content; AD = anthesis date; EPO = ear position; GY = grain yield; PH = plant height; EH = ear height; TEX = grain texture; SC = starch content; OC = oil content.

To understand the interrelations among grain quality traits, Pearson’s correlations of BLUP values were computed within the F_3_ pop 2 (Figure 2). Grain protein content was negatively correlated with starch content (r = −0.56**), grain yield (r = −0.16**) and plant height (r = −0.13*). In the same population, grain yield had a weak but significant positive correlation with grain texture (r = 0.14*), plant height (r = 0.21**) and ear height (r = 0.19**). Grain oil content was negatively correlated with starch content (r = −0.65**). Grain texture and anthesis date were also negatively correlated (r = −0.18**). The documented correlations between grain quality and associated traits in diverse maize populations present valuable insights for making informed decisions in genotypic selection. These findings suggest a complex trait architecture, wherein grain quality traits display significant interactions (+/−) with one another.

### 3.2 QTLs associated with grain quality traits

The maize populations evaluated in this study were also tested in earlier studies (Gowda et al., 2018; Kimutai et al., 2023), where the linkage maps information is included. In brief, the map length of each population was 2,970, 1,650.28, 906.83, and 2,169.97 cM from 1,007, 452, 202, and 387 polymorphic SNPs for DH pop 1 and F_3_ pop 2, 3, and 4, respectively. Across the ten maize chromosomes, a total of 63 significant QTLs were identified for oil content (13), grain protein content (7), starch content (33), grain texture (8) and kernel weight (2) (Table 3). The identified QTLs were distributed across 10 chromosomes. Table 3 contains comprehensive information about the identification and locational specifics of the discerned QTLs, as well as their respective genetic effects. QTLs for oil content were found in all chromosomes except chromosomes 2, 7 and 10. For protein content, QTLs were only discovered on chromosomes 1, 2, 3, 5 and 6. QTLs associated with starch content were found in all chromosomes except on chromosomes 9 and 10. For grain texture, only chromosomes 6 and 10 had no QTLs. For kernel weight, two QTLs on chromosomes 4 and 10 were found in F_3_ pop3.

**TABLE 3:** Analysis of markers associated with grain quality traits, allele substitution (α) effects, and the total phenotypic variance of the joint linkage association mapping based on combined DH and F3 maize populations.

QTL name	Chr	Position (cM)	LOD	PVE (%)	Add	Dom	Total PVE (%)	Flanking markers		Physical position (Mbp)
DH pop 1 - CML494×CML550										
Oil content										
*qOC3-146*	3	150	6.3	18.06	0.05	-	30.72	S3_145603187	S3_149385251	145.60–149.38
*qOC6-102*	6	50	8.8	26.85	0.06	-		S6_101991919	S6_103359585	101.99–103.35
*qOC8-16*	8	197	2.98	8.79	−0.03	-		S8_15877080	S8_19122987	15.87–19.12
*qOC9-10*	9	48	3.47	9.58	0.03	-		S9_8416672	S9_10276690	8.41–10.27
**Grain protein content**										
*qGPC9-144*	9	162	2.95	11.88	−0.02	-	10.75	S9_143801329	S9_149382254	143.80–149.38
Starch content										
*qSC10-20*	10	68	2.81	7.44	−0.02	-	9.75	S10_11602996	S10_38530632	11.60–38.53
F3 pop 2 - CKL5017×CML536										
Oil content										
*qOC1-64*	1	165	3.84	7.67	−0.06	0.04	15.53	PZA00455.14	PHM 1968.22	63.80–168.71
*qOC6-74*	6	59	4.59	7.13	−0.06	0.02		PZB01009.1	PZA00942.2	72.95–89.81
*qOC6-90*	6	70	3.74	5.6	0.05	0		PZA00942.2	lac1.3	89.81–112.90
Protein content										
*qGPC1-105*	1	159	5.94	9.14	−0.14	0	17.61	PZA00939.1	PZA00455.14	104.96–168.71
*qGPC2-227*	2	46	2.74	6.19	−0.02	−0.16		PZD00022.5	PZA02266.3	226.34–233.61
*qGPC2-10*	2	207	3.42	4.89	0.09	0.04		PHM6111.5	PZA00613.22	3.50–21.42
*qGPC6-30*	6	28	3.05	19.45	−0.17	−0.04		PZD00072.2	PZA00440.1	21.59–71.99
Starch content										
*qSC1-20*	1	16	5.6	22.46	0.04	0.1	17.96	PHM11114.7	PZA01456.2	12.76–157.65
*qSC1-105*	1	158	3.84	0	0.01	0		PZA00939.1	PZA00455.14	104.96–168.71
*qSC1-21*	1	303	3.59	8.81	−0.03	−0.3		PHM574.14	PZA02094.9	15.33–59.27
*qSC2-189*	2	1	2.96	2.82	0.02	−0.01		PZA01552.1	PZD00022.5	188.45–233.61
*qSC2-194*	2	77	3.11	2.98	0.03	0.01		PZA02964.7	PHM14412.4	193.67–201.69
*qSC2-189*	2	79	3.2	2.2	0.03	0.02		PHM7953.11	PZA00803.3	189.50–189.60
*qSC3-171*	3	20	2.55	4.5	0.04	0.03		PZA03735.1	PHM17210.5	170–91 - 173.26
*qSC3-121*	3	38	3.28	0.1	0.03	0.01		PHM1745.16	PZA00363.7	120.53–129.09
*qSC3-40*	3	44	9.03	4.47	0.04	0		PZA00707.9	PZA00380.10	38.20–98.45
*qSC3-38*	3	47	2.67	0.85	−0.03	0.01		PHM2343.25	PZA00297.2	26.85–38.83
*qSC4-10*	4	113	2.61	10.55	0.01	0.07		PZA02289.2	PZA00436.7	6.55–177.80
*qSC4-20*	4	143	3.92	6.17	−0.04	0.01		PHM259.11	PZA00704.1	16.30–114.90
*qSC5-10*	5	11	3.5	12.99	0.03	−0.2		PHM5484.22	PHM3137.17	8.18–20.83
*qSC5-2*	5	55	2.97	3.72	−0.03	0.02		PZA00963.3	PZA00818.1	1.0–87.11
*qSC5-119*	5	113	3.15	4	−0.03	−0.02		PZA02164.16	PZA01365.1	117.35–138.85
*qSC6-90*	6	60	5.14	2.56	0.03	−0.01		PZA00942.2	lac1.3	89.81–112.90
*qSC6-113*	6	71	5.18	0.81	−0.03	0		lac1.3	PZA00571.1	112.90–113.26
*qSC6-125*	6	88	5.05	3.39	−0.04	0.05		PZB00414.2	PZA02328.5	124.71–130.59
*qSC7-15*	7	35	4.82	2.44	0.02	0		PZB00752.1	PZA02872.1	11.07–113.74
*qSC8-10*	8	9	9.85	1.24	0.01	0		PHM2487.6	PZA02955.3	8.40–15.16
*qSC8-105*	8	39	5.65	1.93	0	0.03		PZA02566.1	PHM934.19	104.22–109.38
*qSC8-123*	8	47	3.08	5.06	0.02	0.06		PZA01049.1	PHM4757.14	122.03–144.66
Grain Texture										
*qGT1-43*	1	157	4.5	4.76	0.06	−0.03	40.55	PZA00962.1	PZA00939.1	42.32–104.96
*qGT1-55*	1	304	2.53	5.52	−0.3	−0.53		PHM574.14	PZA02094.9	15.33–59.27
*qGT3-9*	3	68	11.5	14.59	0.11	−0.03		PZA00508.2	PZA01765.1	8.86–11.33
*qGT4-10*	4	123	7.21	8.03	−0.08	−0.02		PZA02289.2	PZA00436.7	6.55–177.80
*qGT5-88*	5	112	6.33	11.29	0.1	0.02		PZA01693.1	PZA02164.16	87.11–117.35
*qGT7-82*	7	91	2.73	7.71	−0.02	0.56		PZA01933.3	PHM3435.6	80.61–141.80
*qGT9-87*	9	22	2.76	3.49	0.06	0		PHM1766.1	PZA03235.1	86.54–107.26
*qGT9-80*	9	32	6.68	7.06	−0.08	0		PZA03235.1	PZA00225.8	76.31–86.54
F3 pop 3 - CML494×CML550										
Oil content										
*qOC3-06*	3	77	5.53	3.51	0.04	0	15.53	PZA01765.1	PHM12859.7	5.16–8.86
*qOC5-05*	5	78	3.47	2.49	0.03	0.01		PHM14671.9	PHM3096.19	3.83–5.50
Grain protein content										
*qGPC3-175*	3	30	2.89	4.21	0.1	−0.04	17.61	PZA00538.15	PHM17210.5	170.91–199.68
*qGPC5-10*	5	53	5.23	6.16	−0.12	0.03		PZA03340.2	PHM14671.9	5.50–19.33
Starch content										
*qSC1-227*	1	38	3.79	2.38	0.02	0.02	17.96	PHM5293.11	PZA00381.4	226.24–232.45
*qSC1-192*	1	67	2.56	3.57	0.04	0.03		PHM5480.17	PZA00425.11	191.75–209.98
*qSC3-175*	3	32	7.8	1.19	−0.03	0		PZA00538.15	PHM17210.5	170.91–199.68
*qSC3-06*	3	76	3.6	3.57	−0.04	0.02		PZA01765.1	PHM12859.7	5.16–8.86
*qSC5-70*	5	49	11.2	2.38	0.02	−0.01		PHM13675.18	PHM 1870.20	61.25–71.08
*qSC5-35*	5	51	13.8	1.19	0.02	−0.02		PZA01530.1	PZA00934.2	31.50–39.24
*qSC6-121*	6	65	3.01	1.19	−0.02	−0.01		PZA00473.5	PZA02673.1	117.06–135.75
*qSC8-10*	8	16	3.64	1.19	0.01	−0.03		PHM2487.6	PZA03178.1	8.41–11.88
Kernel weight										
*qKw4-170*	4	62	3.02	4.54	−0.31	0.10	9.27	PHM3155.14	PZA01187.1	168.25–175.15
*qKwt10-100*	10	1	3.84	4.50	0.33	0.05		PZB01358.1	PHM229.15	92.58–107.42
F3 pop 4 - VL081452×VL058589										
Oil content										
*qOC2-185*	2	207	11.6	25.93	−0.011	0.64	15.53	PZA02170.1	PHM5060.12	184.40–231.77
*qOC4-230*	4	89	3.26	5.98	0.03	0.001		PZA00521.3	PZA01367.2	226.98–243.38
*qOC4-170*	4	126	3.67	6.74	0.041	−0.01		PHM 2006.57	PZA01289.1	167.40–171.71
*qOC6-70*	6	126	3.78	6.97	0.037	−0.03		PZA00006.17	PHM14522.5	66.16–73.59
Starch content										
*qSC5-55*	5	196	3.65	19.74	0.095	−0.46	19.81	PZA02207.1	PHM2769.43	51.25–59.62
*qSC7-121*	7	43	2.5	7.34	−0.091	0.201		PHM9162.135	PZA02959.14	120.22–133.75

Chr–chromosome; PVE, phenotypic variance explained; add–additive effects; Dom–dominance effects.

In DH pop1, QTL analysis revealed a total of 6 QTLs for the studied grain quality traits on chromosomes 3, 6, 8, 9 and 10 (Table 3). The highest number of QTLs were discovered for starch content in F_3_ pop 2 (n = 22). For the same population, 8 and 4 QTLs discovered were associated with grain texture and protein content respectively. No QTLs were detected for protein content in F_3_ pop 4. A comparison of the QTLs across DH and F_3_ populations revealed that several QTLs overlapped for some of the grain quality traits. In Chromosome 6, the region between 89.81 and 112.9 Mb had QTLs for both oil and starch content. In chromosome 2, QTLs associated with protein content and starch content were detected in the region between 188.45 and 233.61 Mb (Table 3).

The phenotypic variance explained (PVE) for all detected QTLs associated with grain quality traits ranged from 0.81% to 26.85% (Table 3). The most extensive range of PVE was observed in F_3_ pop 2 (0.81%–22.46%) for starch content. On the other hand, a narrow PVE range was observed for oil content (2.49%–3.51%) in F_3_ pop 3. Significant QTLs that explained more than 10% of the PVE (major effects), was identified for oil content (*qOC3-146* (18.06%), *qOC6-102* (26.85%), *qOC2-185* (25.93%) and *qOC2-185* (25.93%)), protein content (*qGPC6-30* (19.45%), starch content (*qSC1-20* (22.46%), *qSC4-10* (10.55%), *qSC5-10* (12.99%) and *qSC5-55* (19.75%)) and grain texture (*qGT3-9* (14.59%) and *qGT5-88* (11.29%)). In DH pop1, additive effects are high for two major effect QTL and the favourable alleles are contributed from parent CML550. Whereas for the major effect QTL identified for grain protein content, the favourable alleles are contributed from parent CML494 (Table 3). In F3 pop 2, the favourable alleles for major effect QTL for protein content was contributed from parent CKL5017, whereas for starch content and grain texture, parent CML536 contributed.

### 3.3 Prediction accuracies of grain quality traits in DH and F3 tropical maize populations

**FIGURE 3:**
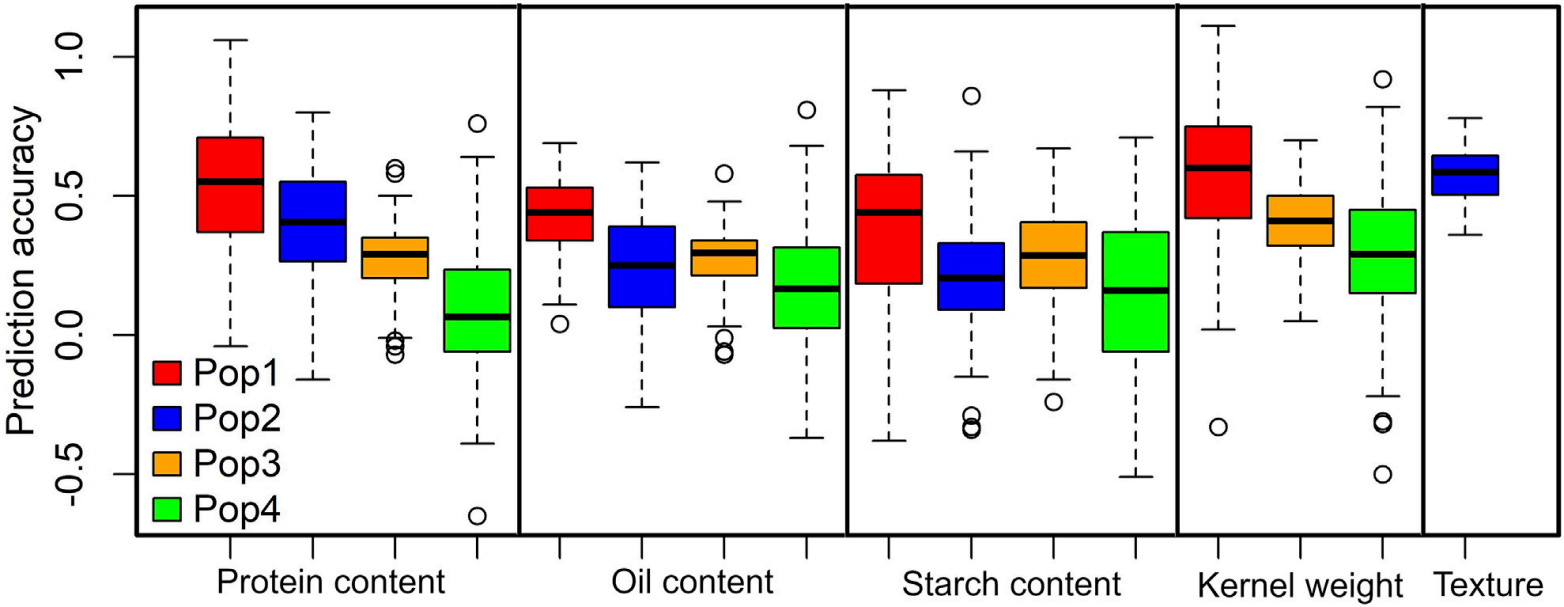
Distribution of the five-fold cross-validated genomic prediction accuracies in DH pop 1, F_3_ pop 2, F_3_ pop 3 and F_3_ pop 4 for grain quality traits.

To estimate the prediction accuracy for each of the studied grain quality traits, we used the RR-BLUP model (Figure 3). Overall, the prediction accuracies across the populations were moderate to high for the studied grain quality traits. The average prediction accuracies were higher for kernel weight in DH pop 1 (0.58) followed by grain texture (0.57). The prediction accuracies for protein content were 0.52, 0.41, 0.28 and 0.08 for DH pop 1, F_3_ pop 2, F_3_ pop 3 and F_3_ pop 4, respectively (Figure 3). For oil content prediction accuracies were 0.43, 0.25, 0.28 and 0.17, whereas for starch content prediction accuracies were 0.38, 0.21, 0.28 and 0.15 in DH pop 1, F_3_ pop 2, F_3_ pop 3, and F_3_ pop 4, respectively. Interestingly, DH pop 1 outperformed F_3_ maize populations in terms of overall trait genomic prediction accuracy for all traits.

## 4 Discussion

### 4.1 The positive and negative correlations among grain quality traits indicate their potential for simultaneous improvement through phenotypic selection

The significant variability observed within the four tropical maize populations for the studied grain quality traits (Figure 1; Table 2) underscores the genetic diversity present within the studied maize genotypes, emphasizing the potential for targeted breeding efforts to capitalize on this diversity and enhance grain quality traits in the subsequent generations. In an earlier study by Ndlovu et al. (2022), a pronounced range of variability in grain quality traits in maize was documented, despite their study being conducted under conditions of low-N-induced stress. In our study, the tested bi-parental maize populations exhibited a typical grain quality trait composition with moderate protein (6.8%–10%) and oil (4.5%–6.2%) levels, and a substantial starch content reaching 72.5%. These values are consistent with the general nutritional profile of maize reported in earlier studies (Dei, 2017; Ray et al., 2019; Álvarez-Iglesias et al., 2021; Ertiro et al., 2022; Langyan et al., 2022; Wang et al., 2023). Targeted breeding could optimize the grain quality traits to meet the requirements of the food and feed industry.

The observed significant genotypic variance for grain quality traits underscores the substantial genetic diversity present within the tropical maize populations, presenting opportunities for trait enhancement through selective breeding. Furthermore, the significant G×E interactions observed for traits like protein and starch content underscore the importance of incorporating environmental factors into breeding strategies for these traits. These findings align with those of Katsenios et al. (2021) and Ndlovu et al. (2022), particularly regarding the grain protein content in maize. The results of these studies indicate that grain quality is influenced by a variety of environmental conditions. In this respect, to enhance grain quality traits, breeders and seed growers should consider cultivating maize lines in optimal environments. Despite the observed significant G×E interactions for these traits, environmental variance across the studied grain quality traits was not significant. This indicates that genetic factors predominantly govern the trait variations, minimizing the role of external environmental conditions.

Broad sense heritabilities for all the studied grain quality traits ranged from low to high, indicating the varying degrees of genetic influence on the trait variability. Grain oil content demonstrated moderate to high heritability, suggesting that a significant portion of the phenotypic variation was attributed to genetic factors, making it feasible for recurrent selection approaches. Conversely, grain protein and starch content exhibited a range of heritability from low to high, indicating a complex interplay of genetic and environmental factors. The significant negative correlations observed among all grain quality traits (Figure 2) indicate an inverse relationship, suggesting that an increase in one of the traits can lead to a decrease in the other trait. This result has implications for trait selection in breeding programs for improved grain quality in tropical maize, highlighting the need to carefully consider trade-offs and prioritize traits based on the set breeding objectives.

### 4.2 Grain quality traits in tropical maize are controlled by multiple QTLs

Grain quality traits in maize are characterized by their complexity as quantitative traits, governed by a combination of both major and minor genetic effects (Zheng et al., 2021). In the present study, we used linkage mapping to identify significant QTLs associated with grain quality traits in DH and F_3_ tropical maize populations grown in Kenya. Numerous studies have extensively explored the genetic underpinnings of maize grain quality traits, resulting in the detection of a multitude of QTL (Li et al., 2009; Guo et al., 2013; Galić et al., 2017; Karn et al., 2017; Badu-Apraku et al., 2020; Ndlovu et al., 2022; Zhang et al., 2023).

In accordance with our results, the presence of QTLs related to oil content across all chromosomes except for chromosomes 2, 7, and 10, indicates a widespread genetic regulation of grain oil content in maize. Similarly, QTLs governing grain protein content were exclusively identified on chromosomes 1, 2, 3, 5, and 6, emphasizing the specific chromosomal regions contributing to protein variations. Furthermore, the near-ubiquitous distribution of QTLs associated with grain starch content across most chromosomes, except for chromosomes 9 and 10, highlights the genetic complexity underlying grain starch content in maize. These findings diverge slightly from those obtained by Zhang et al. (2023), who utilized different genotypes from the ones in our study and detected a combined total of 16 QTLs for grain oil content distributed across all maize chromosomes. Yang et al. (2012) also identified 58 QTLs for kernel oil content in all chromosomes. Interestingly, Zhang et al. (2023) recognized chromosome 9 as housing the largest effect QTLs for oil content. Yang et al. (2012) and Ndlovu et al. (2022), on the other hand, reported QTLs on chromosomes 1 and 2 as having the largest effects on grain oil content, respectively. Another study by Fang et al. (2021) identified five major effect QTLs associated with oil content located on chromosomes 6 and 9. For grain protein content, Lu et al. (2022) found associated QTLs in all chromosomes except chromosomes 6, 8 and 10. In the study by Ndlovu et al. (2022), the only major effect QTL associated with grain protein content was identified on chromosome 3. For starch content, the major effect QTLs on chromosomes 1, 3, 4, 5, 7, 8 and 9 were located in regions which were also reported in earlier studies (Goldman et al., 1993; Yang et al., 2013; Lin et al., 2019; Zhang et al., 2022). The absence of QTLs on certain chromosomes in our study, despite earlier reports, underscores the intricate interplay of genetic variations and environmental factors in QTL identification, likely influenced by the distinct genotypes and growing conditions used. This also highlights the importance of broadening the scope of grain quality trait breeding research to encompass diverse maize populations and environmental settings so as to comprehensively uncover QTLs associated with grain quality traits.

DH pop1 and F_3_ pop3 share common parents. However, the average performance was relatively higher for most of traits in DH population with mean of 5.62%, 8.21%, 70.9% and 65.36 g observed for grain oil content, protein content, starch content and grain weight, respectively, compared to 5.17%, 7.33%, 70.9% and 54.3 g in F_3_ pop3 (Figure 1). The number of QTL detected was also varied between these populations with more QTLs (4) detected for oil content in DH population whereas more QTL for starch content were detected in F_3_ pop3 (Table 3). Interestingly, there is no overlapping QTLs were detected in this study. All QTL detected in F_3_ pop3 are associated with minor effect whereas, with DH population, we able to identify one major effect QTL for grain protein content (*qGPC9-144* with PVE of 11.88%) and two major effect QTL for oil content (*qOC3-146* with PVE of 18.06%; *qOC6-102* with PVE of 26.85%). Overall, DH population has few advantages over F_3_ population both in terms of mean performance and finding QTLs, however, results from different genetic populations are required for appropriate conclusion.

The QTLs identified in the present study were found to overlap with those previously reported in diverse maize populations (Ertiro et al., 2022; Ndlovu et al., 2022), indicating a degree of genetic consistency across different studies. A significant finding of our analysis is the identification of distinct genomic regions on chromosome 6 associated with both oil and starch content (Table 3). The detection of QTLs in the genomic region spanning 89.81–112.9 Mb (chr 6) for these traits holds promise for further improvement of these traits. Furthermore, another distinct region from 188.45 to 233.61 Mb on chromosome 2 was found to harbour QTLs linked to grain protein and starch content. The identified regions on chromosomes 2 and 6 hold significant potential for enhancing grain quality in tropical maize through targeted breeding. Elucidating the underlying genes and mechanisms within these regions could provide invaluable insights for developing effective improvement strategies. In previous investigations, research studies by Wassom et al. (2008) and Fang et al. (2021) highlighted the crucial involvement of chromosome 6 in shaping grain quality traits, further substantiating its importance in genetic studies related to maize grain quality. In a similar context, earlier studies (Ndlovu et al., 2022; Zhang et al., 2022) have identified chromosome 2 as a pivotal genomic region associated with grain quality traits.

In maize, starch content is regulated by many genes (Zhong et al., 2020). Starch synthesis in kernels involves a series of starch metabolic enzymes like sucrose synthase (*SUS*), starch synthases (*SS*s) and starch branching and debranching enzymes (Nelson and Pan, 1995; Zhang et al., 2022). Finding candidate genes within the identified QTL regions improves the consistency of identified QTLs to be used in breeding to improve the linked trait. Two QTLs for starch content on F_3_ pop2 (*qSC1-20* and *qSC1-21*) were overlapped with two QTLs for grain texture (*qGT1-43* and *qGT1-55*) and one QTL each for oil content (*qOC1-64*) and protein content (*qGPC1-105*) (Table 3). By using four DH populations, Zhang et al. (2022) also reported QTL for starch content in the same region, which is known to harbor sucrose synthase gene. *Sus2* is one of the three SUS encoding genes in maize located at 57.45 Mbp on chromosome 1, which has a unique role in cytoplasmic sucrose metabolism (Deng et al., 2020). Two QTLs from F_3_ pop2 for starch content (*qSC4-10* and *qSC4-20*) are co-located with grain texture QTL (*qGT4-10*) Several studies also reported colocalized QTL in the same region (Wang et al., 2010; Guo et al., 2013; Yang et al., 2013; Dong et al., 2015; Hu et al., 2021; Zhang et al., 2022). This region also harbors three candidate genes involved in starch synthesis like vacuolar invertase (*IVR2*) at 69.45 Mbp, Starch branching enzyme (*SBE1*) at 65.19 Mbp and *SS2c* at 34.23 Mbp (Zhang et al., 2022). *IVR2* (Kim et al., 2000) in maize has a key role in carbon metabolism in both source and sink tissues that irreversibly hydrolyze sucrose to fructose and glucose and regulates sugar accumulation in sink organs (Juarez-Colunga et al., 2018). *SBE1* is related to amylose content and starch molecular structure (Zhong et al., 2021). Another candidate gene *SS2c* encodes soluble starch synthases which are responsible for synthesis of amylopectin (Yan et al., 2009). These results suggested that QTLs detected in this study are overlapped between traits and with earlier studies and related to series of candidate genes encoding key enzymes relevant to grain quality traits. Therefore, these QTLs are relevant and useful to be used in breeding through marker-assisted breeding to improve the grain quality in maize.

### 4.3 Genomic selection in maize breeding for improved grain quality can serve as a valuable complement to conventional phenotypic selection methods

Genomic selection (GS) is rapidly gaining prominence in maize breeding programs, enabling the precise prediction of breeding values for individual maize lines (Crossa et al., 2017; Atanda et al., 2021; Singh and Kaundal, 2023). This approach has been extensively employed across diverse maize genotypes in numerous studies (Badji et al., 2020; Beyene et al., 2021; Gowda et al., 2021; Ma and Cao, 2021) to investigate various grain-related traits. Our genomic selection analysis revealed a range of prediction accuracies across the maize populations (Figure 3), indicating the promise of genomic information in predicting and selecting grain quality traits in maize breeding programs. Recorded moderate to high prediction accuracies offer promise in maize breeding for targeted traits (Gowda et al., 2021). Interestingly, our analysis revealed higher prediction accuracies for kernel weight, grain texture, and grain protein content, highlighting their suitability for GS. Additionally, we observed that overall trait prediction accuracy in DH pop 1 was superior as compared to the other three F_3_ populations, indicating the potential for leveraging this DH population for more effective trait prediction and selection. A similar trend was observed by Ndlovu et al. (2022), who reported the highest prediction accuracies in DH populations under low N stress conditions. The differences between maize genotypes in overall trait accuracies underscore the importance of considering population-specific factors such as genetic diversity, population structure and trait variability within population or environmental interactions, which may influence the effectiveness of GS for grain quality traits.

## 5 Conclusion

Here, we utilized DH and F_3_ tropical maize populations to detect significant QTLs associated with grain quality traits in maize lines grown in Kenya. The results of this investigation offer valuable genetic resources for molecular breeding and enhance our understanding of the genetic framework governing grain quality in tropical maize. The detection of multiple QTLs distributed across the 10 maize chromosomes suggests that the regulation of grain quality traits involves a combination of both major-effect and minor-effect QTLs. Notably, there was an observation of QTL overlap for protein, oil and starch contents, suggesting potential genetic links or pleiotropic effects. Moving forward, it is essential to prioritize the validation of these identified QTLs, as they hold the key to enhancing the efficiency of maize breeding programs aimed at improving grain quality within the SSA region. The results of this study also illustrated that integrating GS into tropical maize breeding programs focused on improving grain quality can serve as a valuable complement to conventional phenotypic selection methods.

## Data Availability

The original contributions presented in the study are included in the article/Supplementary Material. The marker data is accessible through https://hdl.handle.net/11529/10548940 and https://zenodo.org/records/10021760.
